# Effects of Temperature and Extraguild Prey Density on Intraguild Predation of *Coccinella septempunctata* and *Harmonia axyridis*

**DOI:** 10.3390/insects16010062

**Published:** 2025-01-10

**Authors:** Xia Wen, Guizhen Gao

**Affiliations:** Research Centre for Green Prevention and Control Technology of Forestry Pest, College of Forestry and Landscape Architecture, Xinjiang Agricultural University, Urumqi 830052, China; wenxia970806@163.com

**Keywords:** *Harmonia axyridis*, *Coccinella septempunctata*, *Chromaphis juglandicol*, temperature, intraguild predation

## Abstract

The ladybirds *Coccinella septempunctata* and *Harmonia axyridis* are important biocontrol agents for the small walnut aphid *Chromaphis juglandicola*, a key walnut pest. *C. juglandicola* outbreaks occur in walnut orchards, and walnut yields have declined. Intraguild predation (IGP) is prevalent among natural enemies that coexist in shared habitats and prey upon the same extraguild prey. We designed laboratory experiments to evaluate the potential for IGP between these two ladybirds at different temperatures and extraguild (EG) prey densities, and the ability of IGP to control EG prey under different conditions. We measured IGP rates in first instar larvae, female adults, and male adults (both starved for 24 h) in the vulnerable immature life stages of two ladybird eggs. Intraguild (IG) prey (*H. axyridis* eggs or *C. septempunctata* eggs) and EG prey (*C. juglandicola*) consumption were tallied after 24 h. Temperature and EG prey density influenced IGP rates, with temperature contributing the most to the variance. IGP increased with increasing temperature (15–35 °C), with both factors interactively influencing the EG prey consumption rate and exhibiting highly significant effects. EG prey consumption increased with temperature and density. This research provides theoretical support for the rational use of *H. axyridis* and *C. septempunctata* in the joint biological control of *C. juglandicola*

## Simple Summary

*Chromaphis juglandicola* is a serious insect pest of walnut trees. *Harmonia axyridis* and *Coccinella septempunctata* are ladybird species that are important predators of *C. juglandicola*. This study aimed to determine the effects of extraguild prey density and temperature on intraguild predation (IGP) between two ladybird species. The results showed that IGP increased with increasing temperature (15–35 °C) and decreasing extraguild prey density. The number of extraguild prey consumed by *C. septempunctata* or *H. axyridis* increased with increasing temperature. These results provide a scientific basis for the rational use of *H. axyridis* and *C. septempunctata* to control small walnut aphids.

## 1. Introduction

Intraguild predation (IGP) refers to predation that occurs when predators at the same trophic level compete for similar or identical prey [1,2,3]. Previous studies have shown that ladybird IGP tends to occur with those sharing the same habitat and extraguild (EG) prey [3,4]. *Harmonia axyridis* (Pallas, 1773) and *Coccinella septempunctata* (Linné, 1758) are dominant natural enemies in the Yili region of Xinjiang [5]. They belong to the order Coleoptera and family Coccinellidae, and they are two common and important predatory insects in agricultural and forestry ecosystems [6,7,8]. *H. axyridis* (whether invasive or naturally occurring) is detrimental to other ladybird species; it has a voracious appetite that enables it to outcompete and even consume other ladybird species [6]. Due to their strong predation ability, wide feeding range, and adaptability, these two species are widely used in the biological control of certain pests such as aphids [9,10] and whiteflies [11,12] in various regions. With the promotion of biological control, an increasing number of natural enemy insect combinations are being used in biological pest control [13,14]. It is known that IGP may be affected by a variety of external factors, such as temperature and EG prey density [15,16,17,18], and that IGP is negatively correlated with EG prey density [16] and positively correlated with temperature [17]. However, few studies have reported the effects of temperature and EG prey density on IGP between *H. axyridis* and *C. septempunctata*.

The walnut tree pest, *Chromaphis juglandicola* (Kaltenbach, 1843) (Hemiptera: Drepanosiphidae), is a key pest of cultivated walnut trees in China and several other countries [19,20,21,22]. Typically, *C. juglandicola* populations increase rapidly immediately after sprouting and growing leaves in early spring, and they remain active until the walnuts mature. The pest is distributed on the back of walnuts, and it harms walnut leaves by piercing and sucking. A large number of *C. juglandicola* nymphs and adults can cause fungal contamination and leaf shedding and affect the normal growth of walnut fruits [23,24]. Continuous damage can cause a loss of more than 25% in production [25]. Aphids belong to the r-class of strategic insects and are characterised by strong concealment and short developmental duration [26,27,28]. Farmers often use chemical pesticides for the fast prevention and control of aphid populations; however, their irrational use can cause the pests to develop resistance [29,30,31]. In addition, their use decreases the abundance of natural enemies, which makes applying more pesticide products necessary [32,33,34]. The interaction between pesticides and natural enemies is an important factor driving integrated pest management (IPM), and protecting the natural enemies of insects is beneficial for the biological control of pests.

Both *H. axyridis* and *C. septempunctata* currently contribute to the biological control of *C. juglandicol* [19,20]. As an important influence on the physiological and behavioural changes in insects, temperature plays a crucial role in the occurrence, development, feeding, and reproduction of ladybirds [35]. We hypothesised that temperature affects the IGP of ladybirds. Here, we aimed to evaluate how the temperature and density of EG prey (*C. juglandicol*) affect the IGP of these two ladybirds. To this end, we measured the IGP of *H. axyridis* and *C. septempunctata* eggs in a vulnerable immature life stage using first instar larvae, adult females, and male adults. We aimed to determine whether IGP is affected by temperature and EG prey density in order to speculate on how it might affect the biological control capabilities of *H. axyridis* and *C. septempunctata*.

## 2. Materials and Methods

### 2.1. Insect Rearing

Adults of *C. septempunctata* and *H. axyridis* were collected from a walnut orchard in Xinyuan County, Yili Kazak Autonomous Prefecture (43°27′00′′ N, 83°34′25′′ E) from May to June 2023, and transferred to ventilated plastic containers (7.5 cm × 7 cm × 4 cm) with moisturising filter paper in a ratio of 1:2 between males and females for mating, one container for each species. They were held in a climate-controlled growth chamber set to 25 ± 1 °C, L:D = 14:10 h; and 65 ± 10% RH. To reduce any possible effects of prey conditioning, both the adults and larvae were fed with *Acyrthosiphon pisum* (Harris, 1776). Each beetle pair received sufficient *A. pisum* and was sealed with gauze and a leather band, both of which had the prey replaced daily. Eggs were laid by females directly on cups or oviposition paper and harvested by simply transferring the beetles to a new dish. Part of the harvested eggs were stored in a refrigerator at 4 °C for subsequent experiments. The other part of the eggs hatched, and the newly obtained larvae were transferred to clean Petri dishes (90 mm diameter × 15 mm height) and provisioned with sufficient *A. pisum* ad libitum, refreshed daily until pupation.

*Acyrthosiphon pisum* was collected from broad bean in Xinyuan, China, and reared under the same conditions as the beetles. The aphid colony was reared on *V. faba* plants sown in plastic flowerpots (35 cm height × 20 cm diameter) containing soil. Plants were grown at room temperature and infested with aphids when they had five or more true leaves for subsequent cultivation of the two ladybird species. The branches of walnut leaves infected with *C. juglandicola* were covered and tied tightly using a 60 cm × 45 cm nylon mesh bag (100 mesh) and cultivated. Fresh walnut leaves that were not infested with *C. juglandicola* and those that were maintained in nylon nets for each experiment were used within 12 h of collection. All experiments were conducted in Petri dishes (90 mm diameter × 15 mm height). In each experiment, *C. juglandicola* was the extraguild (EG) prey and *H. axyridis* or *C. septempunctata* eggs were the intraguild (IG) prey. They were presented on walnut leaves, with the leaves placed in Petri dishes containing moist filter paper on the bottom.

### 2.2. Effects of Temperature and EG Prey Density on IGP and EG Prey Consumption

To test the effects of temperature and EG prey density on the IGP between *H. axyridis* and *C. septempunctata*, the EG prey densities of 2nd/3rd instar nymphs were determined on a walnut leaf disc placed in a Petri dish (90 mm diameter × 15 mm height) with the based lined with moist filter paper. To standardise hunger levels, both the first larvae and adults were isolated in Petri dishes (90 mm diameter × 15 mm height) and starved for 24 h. When the predator was a first instar larva, the prey densities were 0, 3, and 9 aphid nymphs of EG prey, and the IG prey densities were 10 eggs. When the predator was a female or male adult, the prey densities were 0, 50, and 500 aphid nymphs of EG prey, and IG prey densities were 100 eggs. Therefore, a total of 45 treatments were studied for each ladybird species, and seven individual beetles were employed as replicates (Table 1). After adding the prey, one first larva or adult female or male of *H. axyridis* or *C. septempunctata* starved for 24 h was added and confined to the Petri dish. The Petri dishes were placed in environmental chambers under five different constant temperatures—15, 20, 25, 30, and 35 ± 1 °C, with 65 ± 10% RH and a light/dark photoperiod of 14:10 h. The numbers of aphids and eggs consumed by *C. septempunctata* or *H. axyridis* were recorded after 24 h.

### 2.3. Data Analysis

The data were subjected to a two way factorial ANOVA using GLM in SPSS (SPSS25), with temperature and extraguild (EG) prey density as independent factors, and with the consumption of intraguild (IG) prey and EG prey as the response variables. To ensure that the data did not violate the standard normality assumption, we used the Shapiro–Wilk test to examine the predation of ladybirds and perform SQRT transformation on each variable if necessary. The significance of the differences was determined using Duncan’s new multiple-range test, and significant differences were reported at *p* < 0.05. Relationships between the temperature and number of IG prey consumed were described by regression to enable a graphical depiction. Linear, exponential, and logarithmic models were fitted to the data; as a second-order polynomial can product the highest *R*^2^ value, the best fit for the average consumption on IG prey over temperature was given by the second-order polynomial. All statistical analyses were conducted using SPSS version 25.0 (SPSS Inc., Chicago, IL, USA).

## 3. Results

### 3.1. Effects of Temperature and EG Prey Density on IGP

The effects of temperature on IGP within both ladybird groups were highly significant (*p* < 0.0001; Table 2). Under the condition of temperature change, EG prey density had a significant impact only on the IGP of the male adult of *C. septempunctata*, and the first instar larvae and female adults of *H. axyridis* (*p* < 0.05). The interaction between temperature and EG prey density had no significant effect on IGP. Therefore, temperature was used as the main factor for analysis when both temperature and EG prey density changed.

#### 3.1.1. IGP by First Instar Larvae

**Figure 1:**
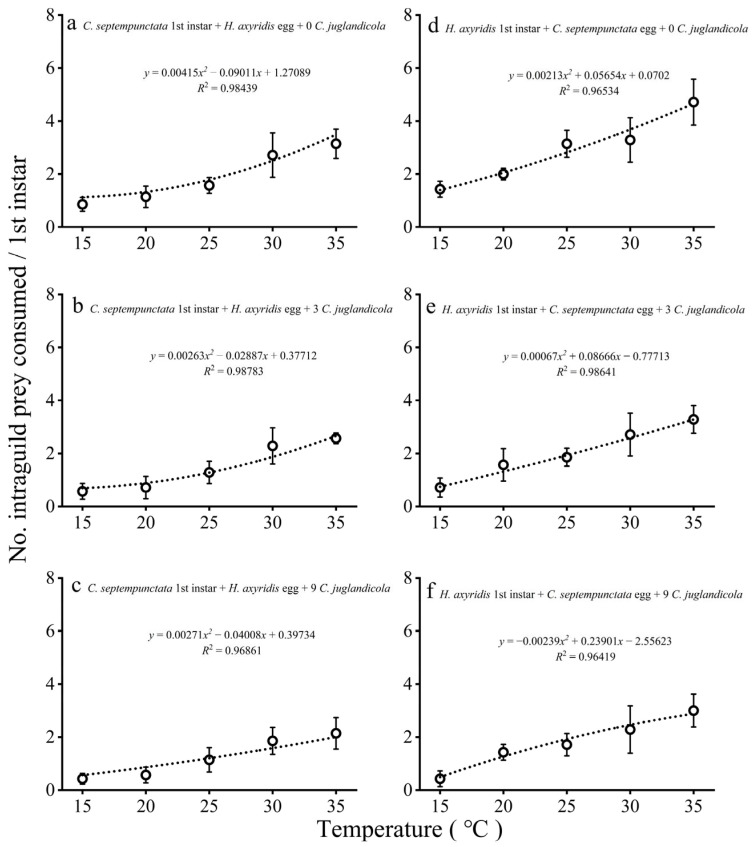
Mean (±SE) numbers of *H. axyridis* eggs (**a**–**c**) and *C. septempunctata* eggs (**d**–**f**) preyed upon by single *C. septempunctata* or *H. axyridis* first instar larva at different temperatures and densities of EG prey, *C. juglandicola*.

The consumption of *H. axyridis* eggs by first instar larvae *C. septempunctata* increased slowly with increasing temperature, and the relationship in all cases was described by second-order polynomial regressions (Figure 1a–c). The IGP of first instar *C. septempunctata* occurred at the highest EG prey offered with increasing temperature.

Consumption of *C. septempunctata* eggs by first instar larvae *H. axyridis* increased slowly with increasing temperature, and the manner in which this occurred bore a striking resemblance to the IGP of *C. septempunctata* first instar larvae (Figure 1d–f). However, IGP at high temperatures generally began at higher absolute levels than IGP in *C. septempunctata*. When the density of EG prey was 0, 3, and 9 per dish, the number of IG prey consumed by *H. axyridis* was 4.71, 3.29, and 3.00, respectively, whereas the number of IG prey consumed by *C. septempunctata* was 3.43, 2.43, and 2.00, respectively.

#### 3.1.2. IGP by Adult Females

**Figure 2:**
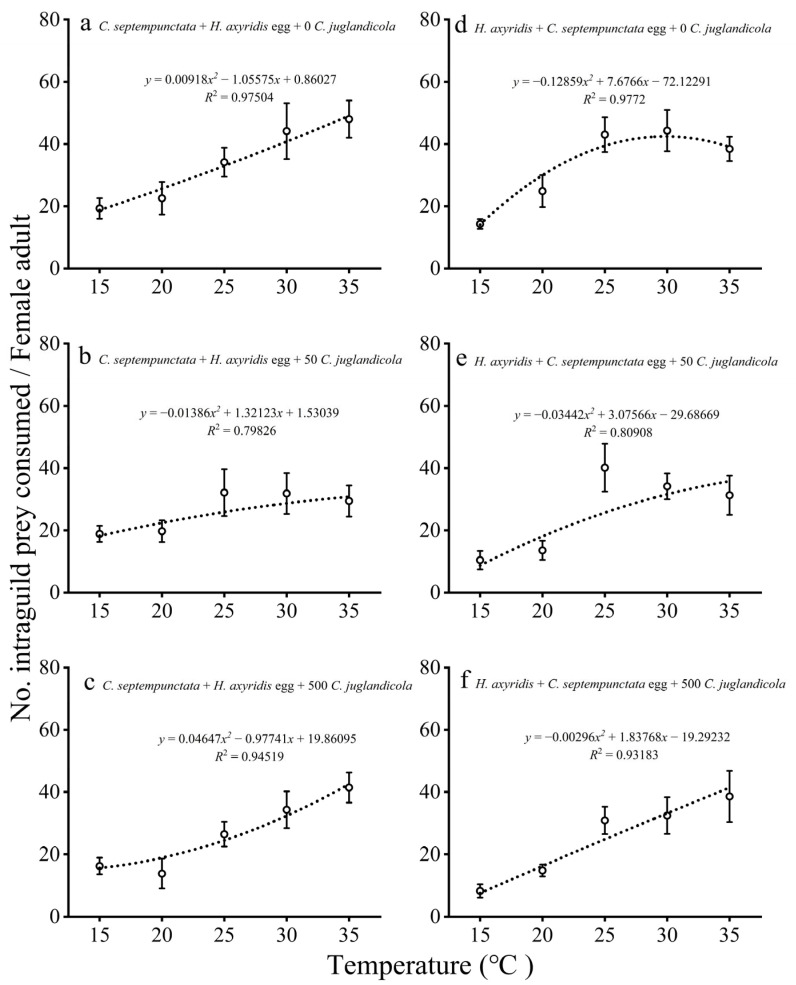
Mean (± SE) numbers of *H. axyridis* eggs (**a**–**c**) and *C. septempunctata* eggs (**d**–**f**) preyed upon by single *C. septempunctata* or *H. axyridis* female adult at different temperatures and densities of EG prey (*C. juglandicola*).

The consumption patterns of *H. axyridis* eggs by adult female *C. septempunctata* also displayed a second-order polynomial relationship with temperature. With an increase in temperature, the IGP of adult female *C. septempunctata* on *H. axyridis* eggs also increased, whereas IGP at high temperatures with low and high densities of EG prey began at generally higher absolute levels than IGP did at low temperatures (Figure 2a,c). However, at an EG prey density of 50, IGP also increased with increasing temperature; however, it first increased and then decreased slowly (Figure 2b).

The consumption patterns of *C. septempunctata* eggs by adult female *H. axyridis* increased with increasing temperature, regardless of whether the densities of EG prey were low or high, in most cases exhibiting a second-order polynomial relationship (Figure 2d–f). Under the condition of insufficient EG prey, the IGP of adult female *H. axyridis* feeding on *C. septempunctata* eggs first increased and then decreased slowly (Figure 2d,e).

#### 3.1.3. IGP by Adult Males

**Figure 3:**
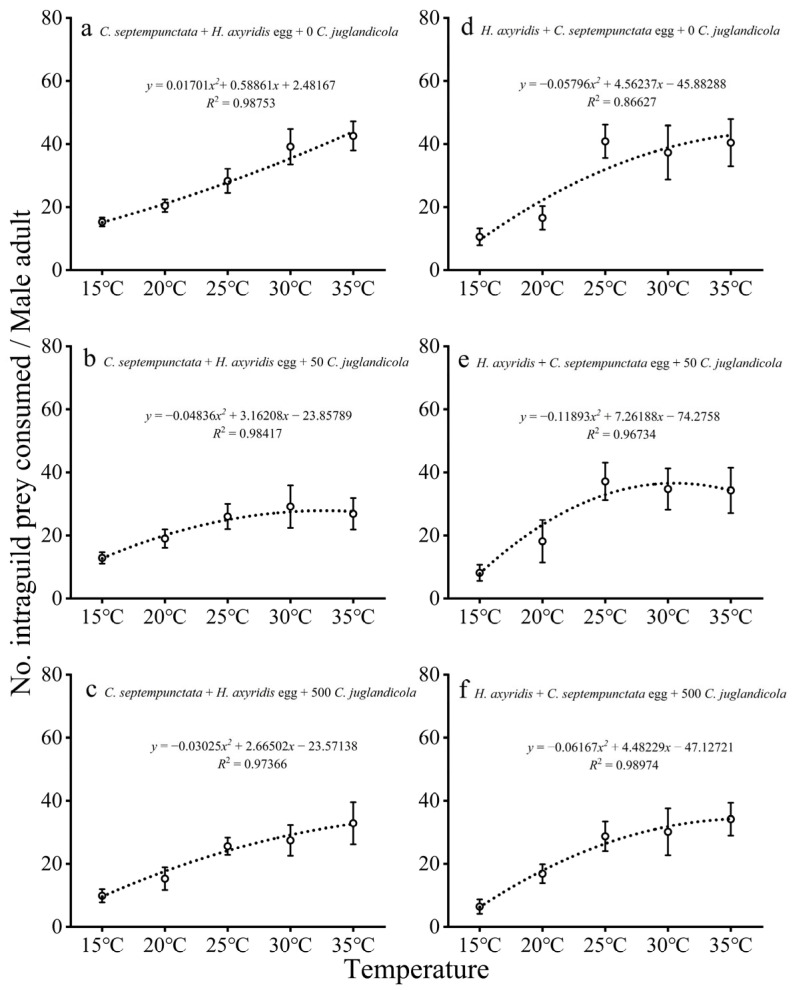
Mean (± SE) numbers of (**a**–**c**) *H. axyridis* eggs and (**d**–**f**) *C. septempunctata* eggs preyed upon by single *C. septempunctata* or *H. axyridis* male adult at different temperatures and densities of EG prey (*C. juglandicola*).

The consumption of *H. axyridis* eggs by male *C. septempunctata* increased with increasing temperature at different EG prey densities, and the relationships in all cases could be determined by second-order polynomial regressions (Figure 3a–c). However, under the conditions of EG prey densities of 50 or 500, the IGP of male *C. septempunctata* first presented an increasing trend and then slowly increased with increasing temperature (Figure 3b,c).

The consumption of *C. septempunctata* eggs by male *H. axyridis* increased substantially with increasing temperature at different EG prey densities, similar to the IGP by *C. septempunctata* (Figure 3d–f). When it reached a certain temperature, the IGP of *C. septempunctata* eggs by male *H. axyridis* increased and slowed down.

### 3.2. Consumption of EG Prey

Two independent factors (temperature and EG prey density) contributed significantly to ladybird beetle EG prey (*p* < 0.0001). The two interaction terms (temperature × EG prey density) were highly significant (*p* < 0.001) (Table 3). Therefore, the predation amount of the two ladybird beetles on EG prey was determined by considering temperature, EG prey, and the two-factor interaction under the condition that both temperature and EG prey density changed.

#### 3.2.1. Consumption of EG Prey by First Instar Larvae

**Figure 4:**
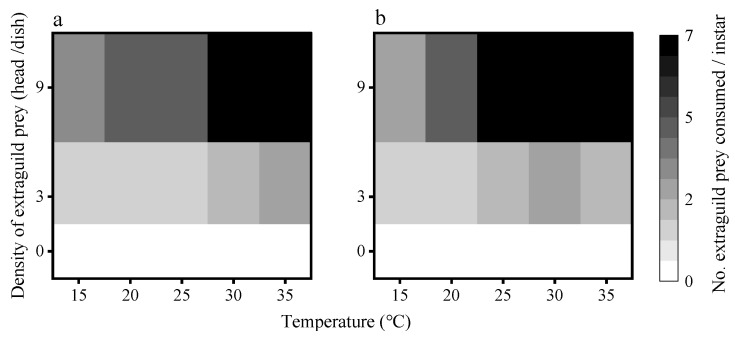
Mean numbers of EG prey (*C. juglandicola*) preyed upon by (**a**) single first instar *C. septempunctata* larva or (**b**) single first instar *H. axyridis* larva at different temperatures and EG prey densities.

Consumption of EG prey by first instar larvae *C. septempunctata* (Figure 4a) increased with rising temperatures (15–35 °C), under the same EG prey density circumstances. Similarly, the predation on EG prey increased with increasing EG prey density under the same temperature conditions. However, when the temperature and density of EG prey increased simultaneously, the consumption of EG prey also increased, reaching a maximum predation capacity of seven heads at the temperature and EG prey density.

Consumption of EG prey by first instar larvae *H. axyridis* (Figure 4b) increased with rising temperatures (15–35 °C), under the same EG prey density circumstances. Similarly to *C. septempunctata*, predation by EG prey increased with increasing EG prey density under the same temperature conditions. The consumption of EG prey was the highest (6.57 heads) at high temperatures.

#### 3.2.2. Consumption of EG Prey by Adults

**Figure 5:**
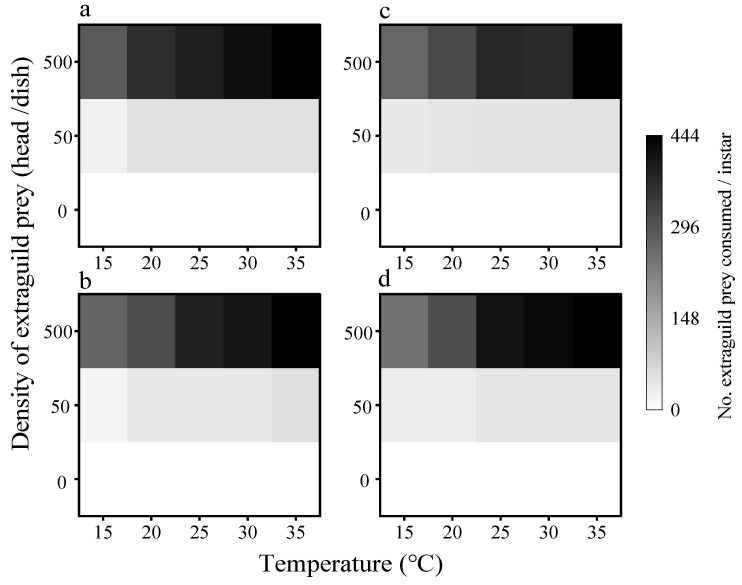
Mean numbers of EG prey (*C. juglandicola*) preyed upon by (**a**) single *C. septempunctata* female adult, (**b**) *C. septempunctata* male adult, (**c**) *H. axyridis* female adult, and (**d**) *H. axyridis* male adult at different temperatures and EG prey densities.

Female and male *C. septempunctata* consumed EG prey at a somewhat higher temperature (35 °C) than the low temperature (15 °C) in all treatments, and a similar situation occurred when only 0, 50, or 500 nymphs were offered together with 100 eggs of *H. axyridis* (Figure 5a,b). Similarly, its predation of EG prey increased with rising EG prey density, under the same temperature conditions. At high temperatures and EG prey, the females and males both consumed the most EG prey at 387.85 and 383.43 heads, respectively.

EG prey consumption by female and male *H. axyridis* (Figure 5c,d) increased with rising temperatures (15–35 °C) under the same density of EG prey circumstances. Similarly to *C. septempunctata,* EG prey predation increased with increasing EG prey density under the same temperature conditions. At high temperatures and EG prey, the females and males both consumed the most EG prey at 444.00 and 404.71 heads, respectively.

## 4. Discussion

Intraguild predation (IGP) is a form of competition and a typical type of predation among various species, the intensity of which affects the population dynamics of each species and the dispersal, survival, or mortality of organisms sharing the same resource [36,37]. Understanding the IGP of *H. axyridis* and *C. septempunctata* under different temperature and EG prey density changes is beneficial for learning about the IGP of these two ladybirds and how they utilise their survival and pest control abilities. Our results showed that both first instar and adult *C. septempunctata* and *H. axyridis* were engaged in IGP and IG prey behaviour (*H. axyridis* eggs or *C. septempunctata* eggs), but the presence of EG prey reduced the IGP between the two species. These results are consistent with those of other studies where the IGP decreased with increased prey density [38,39]. It may be that an increased EG prey dilutes the density of IG prey thereby affecting the search for IG prey by intraguild predators [40,41]. In our study, the consumption of *H. axyridis* eggs by *C. septempunctata* and *C. septempunctata* declined with increasing EG prey densities (*p* > 0.05), while the consumption of EG increased (*p* < 0.05).

These results indicate that temperature significantly influences the predation capacity of *C. septempunctata* and *H. axyridis* for IG prey. We found that the number of IG prey consumed by *C. septempunctata* or *H. axyridis* increased when the temperature increased. This agrees with the findings that *H. axyridis* and *Propylaea japonica* consume more IG prey under increasing temperatures [42], and some studies have found that the frequency is twice as high at high temperatures than at low temperatures [17]. This result may be attributed to the increased activity of natural enemies due to increased temperature, which in turn increases their consumption rates [17,43]. Nevertheless, the IGP between *C. septempunctata* and *H. axyridis* was remarkably symmetric at both temperatures tested, but the EG prey density did not significantly affect the IGP. However, the interaction between temperature and EG prey density was also not significant for the IGP of the two ladybird beetles in this experiment. Therefore, under the condition of simultaneous changes in temperature and EG prey, the effect of temperature on IGP was more significant. This may be due to the fact that temperature can improve the search effect of predators on prey to some extent [44,45,46]. In our study, we found that adults preyed more on groups than larvae, and this may have occurred because adult insects are larger in size and have stronger mobility than larvae. We also found that female adults preyed more than male adults, and this may occur because of their unique physiological characteristics; females require more energy to produce offspring and maintain the dynamic balance of the population [47]. Similar results were also found in other studies [48,49].

As the temperature increased from 15 °C to 35 °C, the consumption of EG prey by first instar larvae, females, and males significantly increased, and the adults consumed more than the larvae. This result likely reflects the fact that the developmental stage can affect the predation of ladybird beetles, and the first instar larvae have the worst predation ability. Kulkarni and Evenden found that *C. septempunctata* increases its attack rates and prey consumption under warmer conditions [50]. Schwarz and Frank also found that *H. axyridis* consumed a significantly greater aphid biomass at higher temperatures [51]. This may be because temperature promotes predator metabolism thereby enhancing the predation ability [52]. Within this temperature range, we found that female adult ladybird beetles of *H. axyridis* and *C. septempunctata* preyed more on EG prey than on male adults, which may be because female adults require more energy to support their physiological functions and reproduce offspring, whereas male adults do not require this additional energy [47].

In conclusion, our study demonstrated that the IGP between *H. axyridis* and *C. septempunctata* was influenced by temperature and EG prey density. Of all the factors, temperature had the strongest effect on the propensity for IGP, whereas the density of EG prey reduced the IGP strength between *H. axyridis* and *C. septempunctata* but had no significant effect. Under IGP conditions, the predation of EG prey by *H. axyridis* and *C. septempunctata* significantly increased with temperature and the density of EG prey. In natural environments, the environmental temperature and density of EG prey, the complexity of their habitat [53], and the hunger level of predators [1] all have an impact on the IGP of natural enemy insects. Therefore, when using *H. axyridis* and *C. septempunctata* to synergistically control *C. juglandicola* under natural conditions, the above factors should be fully considered to indirectly or directly increase IGP to achieve the best biological control effect on the target pests.

## 5. Conclusions

In summary, our study showed that IGP intensity between *H. axyridis* and *C. septempunctata* increased with increasing temperature and decreased with an increase in EG prey density. Therefore, the IGP between *H. axyridis* and *C. septempunctata* under field conditions requires further research to determine their ability to control pests under climate warming conditions. We speculate that if aphids are abundant, *H. axyridis* and *C. septempunctata* could coexist together without affecting their survival or adversely affecting the biological control of aphids.

## Figures and Tables

**Table 1:** Prey treatments provided under different temperatures to individual first instar larvae, female adults, and male adults of *H. axyridis* and *C. septempunctata* as intraguild (IG) predators, using *H. axyridis* or *C. septempunctata* eggs as intraguild (IG) prey and *C. juglandicola* as extraguild (EG) prey.

IG Predator Stage	IG Prey Density	EG Prey Density	Temperature
First instar larvae	10 eggs	0, 3, 9	15 °C, 20 °C, 25 °C, 30 °C, 35 °C
Female adults	100 eggs	0, 50, 500	15 °C, 20 °C, 25 °C, 30 °C, 35 °C
Male adults	100 eggs	0, 50, 500	15 °C, 20 °C, 25 °C, 30 °C, 35 °C

**Table 2:** Results of factorial ANOVA of numbers of intraguild prey *(H. axyridis* eggs, *C. septempunctata* egg) consumed by first instar larvae, female adults, and male adults of *H. axyridis* or *C. septempunctata* when provided with fixed numbers of EG prey (first instar larva: 0, 3, 9; adult: 0, 50, 500) and *H. axyridis* eggs or *C. septempunctata* eggs at different temperatures.

Intraguild Predator	Intraguild Predator Stage	Intraguild Prey Density	Source of Variation	df	*F*	*p*
*C. septempunctata*	First instar larvae		Temperature	4	9.19	<0.0001
		10 *H. axyridis* eggs	EG prey density	2	4.50	0.0052
			Temperature × EG prey density	8	0.26	0.9789
	Female adults		Temperature	4	11.79	<0.0001
		100 *H. axyridis* eggs	EG prey density	2	2.32	0.0918
			Temperature × EG prey density	8	0.29	0.9733
	Male adults		Temperature	4	14.02	<0.0001
		100 *H. axyridis* eggs	EG prey density	2	4.2	0.0080
			Temperature × EG prey density	8	0.64	0.8234
*H. axyridis*	First instar larvae		Temperature	4	10.4	<0.0001
		10 *C. septempunctata* eggs	EG prey density	2	5.48	0.0016
			Temperature × EG prey density	8	0.21	0.9899
	Female adults		Temperature	4	18.71	<0.0001
		100 *C. septempunctata* eggs	EG prey density	2	3.71	0.0118
			Temperature × EG prey density	8	0.50	0.9595
	Male adults		Temperature	4	15.26	<0.0001
		100 *C. septempunctata* eggs	EG prey density	2	1.36	0.2419
			Temperature × EG prey density	8	0.21	0.9895

**Table 3:** Results of factorial ANOVA of numbers of EG prey consumed by first instar larvae, female adults, and male adults of *H. axyridis* or *C. septempunctata* when provided with fixed numbers of EG prey (first instar larva: 0, 3, 9; adult: 0, 50, 500) and *H. axyridis* eggs or *C. septempunctata* eggs at different temperatures.

Intraguild Predator	Intraguild Predator Stage	Intraguild Prey Density	Source of Variation	df	*F*	*p*
*C. septempunctata*	First instar larvae	10 *H. axyridis* eggs	Temperature	4	8.81	<0.0001
			EG prey density	2	184.86	<0.0001
			Temperature × EG prey density	8	4.15	<0.001
	Female adults	100 *H. axyridis* eggs	Temperature	4	9.67	<0.0001
			EG prey density	2	1239.16	<0.0001
			Temperature × EG prey density	8	7.00	<0.0001
	Male adults	100 *H. axyridis* eggs	Temperature	4	10.10	<0.0001
			EG prey density	2	856.15	<0.0001
			Temperature × EG prey density	8	7.39	<0.0001
*H. axyridis*	First instar larvae	10 *C. septempunctata* eggs	Temperature	4	7.71	<0.0001
			EG prey density	2	140.94	<0.0001
			Temperature × EG prey density	8	4.35	<0.0001
	Female adults	100 *C. septempunctata* eggs	Temperature	4	16.97	<0.0001
			EG prey density	2	1506.69	<0.0001
			Temperature × EG prey density	8	14.15	<0.0001
	Male adults	100 *C. septempunctata* eggs	Temperature	4	18.14	<0.0001
			EG prey density	2	1452.06	<0.0001
			Temperature × EG prey density	8	14.22	<0.0001

## Data Availability

The data presented in this study are available upon request; please contact the corresponding author.
